# Association between gestational weight gain and chronic disease risks in later life

**DOI:** 10.1038/s41598-023-50844-4

**Published:** 2024-01-05

**Authors:** Yuki Kamihara, Kohei Ogawa, Naho Morisaki, Naoko Arata, Seiji Wada

**Affiliations:** 1 Center for Maternal-Fetal, Neonatal and Reproductive Medicine, National Center for Child Health and Development, 2-10-1 Okura, Setagaya-Ku, Tokyo, 157-8535 Japan; 2 Department of Social Medicine, National Research Institute for Child Health and Development, Tokyo, 157-8535 Japan

**Keywords:** Weight management, Risk factors

## Abstract

To assess the association between gestational weight gain (GWG) and the risk of developing chronic diseases in later life, this historical cohort study of 318 non-overweight women was conducted between April 2017 and November 2020 at a hospital in Tokyo. Data about GWG in the last pregnancy and the development of any chronic diseases of the subjects were retrieved from the women’s Maternal and Child Health Handbooks and through a questionnaire survey, respectively. The outcomes were chronic diseases, such as diabetes mellitus (DM), hypertension, hyperlipidemia, and being overweight (body mass index [BMI] ≥ 25 kg/m^2^). Association between GWG and outcomes were assessed using a logistic regression analysis.

There were significant positive linear associations between GWG and the risks of developing DM, hypertension, and being overweight (*P* = 0.013, 0.050, and 0.017, respectively). After adjusting for later-life BMI, a significant association between GWG and DM (*P* = 0.025) remained, but the association between GWG and hypertension disappeared. GWG was significantly associated with DM, hypertension, and being overweight later in life. Although the association between GWG and DM was partially independent of BMI later in life, the association between GWG and hypertension was influenced by being overweight later in life.

## Introduction

Recent studies have demonstrated that women with gestational diabetes mellitus (GDM) or hypertensive disorders in pregnancy (HDP) have a higher risk of developing chronic diseases, such as diabetes mellitus (DM), hypertension, hyperlipidemia, and cardiovascular disease, in later life^1,2^. Many studies have demonstrated that excessive gestational weight gain (GWG) during pregnancy is strongly associated with increased risks of GDM^3,4^ and HDP^5,6^. Additionally, some reports have shown that excessive GWG has a significant effect on postpartum weight retention and long-term obesity^7–9^, possibly leading to several chronic diseases. Considering the evidence, avoiding excessive GWG during pregnancy may help improve later-life pregnancy outcomes and health. Thus, epidemiological studies on the association between GWG itself and chronic disease in later life should be crucial because GWG is a representative changeable factor during pregnancy, but to date, none have been conducted.

In Japan, an objective and reliable official recording system for health conditions during pregnancy, “The Maternal and Child Health Handbook” (MCHH) is widely used. The MCHH data should be reliable and useful for conducting epidemiological studies on health conditions during pregnancy as variables of interest^10^. This study used MCHH data to investigate the association between GWG and the risks of developing later-life chronic diseases, such as DM, hypertension, and hyperlipidemia.

## Results

Of the 413 study participants, 87 women with missing data on GWG during their pregnancy period in the MCHH were excluded. Of the remaining participants, five were additionally excluded because of missing data on age at delivery (n = 3) and parity (n = 2). Women with a prepregnancy BMI ≥ 25 kg/m^2^ (n = 3) were also excluded. Finally, the remaining 318 women were included in the primary analysis.

The characteristics of the women are shown in Table 1. The mean duration from delivery to completing the questionnaire was 35.9 years (standard deviation [SD]: 4.2 years), and the mean current age of women was 64.3 years (SD: 5.2 years). Insufficient GWG, adequate GWG, and excessive GWG were observed in 113 (35.5%), 85 (26.7%), and 120 (37.7%), respectively, and DM, hypertension, and hyperlipidemia were present in 19 (6.0%), 86 (27.0%), and 115 (36.1%) women, respectively.

**Table 1:** Characteristics of women (n = 318).

Variables			Mean (SD) or N (%)
Pre-pregnancy BMI and GWG during pregnancy			
	Pre-pregnancy BMI (kg)	< 18.5	63 (19.8)
		18.5–25	255 (80.2)
	GWG during pregnancy (kg)	Insufficient	113 (35.5)
		Adequate	85 (26.7)
		Inadequate	120 (37.7)
Other demographics			
	Current age		64.3 (5.2)
	Age at delivery		28.5 (3.4)
	Primiparity		162 (50.9)
	Height		157 (4.5)
	Current BMI		21.8 (2.8)
	Prepregnancy BMI		20.0 (1.8)
	GWG during pregnancy	11.3 (3.2)	
Current medical conditions			
	Diabetes mellitus	19 (6.0)	
	Hypertension	86 (27.0)	
	Hyperlipidemia	115 (36.1)	
Other variables			
	Duration from delivery to completing the questionnaire	35.9 (4.2)	

BMI: Body mass index.

GWG: Gestational weight gain.

The association of women’s GWG in their pregnancy period with chronic diseases is shown in Table 2. First, a multivariate logistic regression analysis was performed to evaluate the association between GWG and the outcomes, considering women with adequate GWG as the reference. Women’s age at delivery, parity, prepregnancy BMI, height, and the duration from delivery to questionnaire completion were considered as possible confounding factors (Model 1)^10^. Compared with the risk for women with adequate GWG, the risk of all outcomes of interest for women with excess GWG and insufficient GWG were not significantly different. However, there were significant positive linear associations between each GWG category and the risk of DM (*P* for trend: 0.013), hypertension (*P* for trend: 0.050), and of being overweight (*P* for trend: 0.017). There was no significant linear association between GWG and hyperlipidemia. We then conducted a further logistic regression analysis additionally adjusted for current BMI to determine if the associations were mediated by current BMI (Model 2). Although a significant positive linear association between each GWG category and the risk of DM was observed (*P* for trend: 0.025), a significant association between any GWG category and the risk of hypertension disappeared (*P* for trend: 0.398; not significant).

**Table 2:** Association of women’s gestational weight gain with chronic diseases in later life (n = 318).

	Current health conditions														
	Diabetes mellitus				Hypertension				Hyperlipidemia				Overweight^a^ (BMI > 25 kg/m^2^)		
	PR	Crude OR	Adjusted OR^b^ (Model 1)	Adjusted OR^c^ (Model 2)	PR	Crude OR	Adjusted OR^b^ (Model 1)	Adjusted OR^c^ (Model 2)	PR	Crude OR	Adjusted OR^b^ (Model 1)	Adjusted OR^c^ (Model 2)	PR	Crude OR	Adjusted OR^b^ (Model 1)
Insufficient GWG	1/113 (0.9)	0.12 (0.01–1.00)	0.12 (0.01–1.02)	0.12 (0.01–1.04)	24/113 (21.2)	0.82 (0.42–1.60)	0.82 (0.41–1.64)	0.87 (0.43–1.76)	45/113 (39.8)	1.35 (0.75–2.43)	1.37 (0.75–2.51)	1.50 (0.80–2.80)	11/112 (9.8)	0.77 (0.31–1.92)	0.65 (0.24–1.74)
Adequate GWG	6/85 (7.1)	Ref	Ref	Ref	21/85 (24.7)	Ref	Ref	Ref	28/85 (32.9)	Ref	Ref	Ref	10/81 (12.4)	Ref	Ref
Excess GWG	12/120 (10.0)	1.46 (0.53–4.07)	1.36 (0.47–3.93)	1.21 (0.40–3.64)	41/120 (34.2)	1.58 (0.85–2.94)	1.51 (0.80–2.86)	1.15 (0.59–2.23)	42/120 (35.0)	1.10 (0.61–1.97)	1.03 (0.57–1.88)	0.93 (0.50–1.73)	22/115 (19.1)	1.68 (0.75–3.77)	1.84 (0.78–4.38)
		P for trend: 0.007	P for trend: 0.013	P for trend: 0.025		P for trend: 0.027	P for trend: 0.050	P for trend: 0.398		P for trend: 0.451	P for trend: 0.316	P for trend: 0.115		P for trend: 0.045	P for trend: 0.017

GWG: Gestational weight gain during pregnancy, OR: Odds ratio, PR: Prevalence, Ref.: Reference.

a: The association between GWG and overweight was assessed on the basis of 308 women; 10 were excluded because of missing data on current BMI.

b: Adjusted by height, prepregnancy body mass index, parity at pregnancy, age at delivery, and period from delivery to current status (Model 1).

C: Adjusted by height, prepregnancy body mass index, parity at pregnancy, age at delivery, period from delivery to current status, and women’s current BMI (Model 2).

We also conducted a sensitivity analysis considering absolute GWG as exposure instead of categorized GWG in the main analysis. For this sensitivity analysis, we categorized women into quintiles according to GWG: Q1 was the lowest and Q5 was the highest, with Q3 considered the reference group. In this sensitivity analysis, the association between quintiles of GWG and outcomes are shown in Table 3. Similar trends to those in our primary analysis were found.

**Table 3:** Association of women’s absolute gestational weight gain with current chorionic diseases. (sensitivity analysis; n = 318).

	Diabetes melitus			Hypertension			Hyper lipidemia			Overweight^a^ (BMI > 25 kg/m2)		
GWG	PR	Crude OR	Adjusted OR^b^	PR	Crude OR	Adjusted OR^b^	PR	Crude OR	Adjusted OR^b^	PR	Crude OR	Adjusted OR^b^
Q1 (GWG < 8.6 kg)	1/64 (1.6)	0.20 (0.02–1.79)	0.17 (0.02–1.66)	16/64 (25.0)	0.82 (0.38–1.76)	0.82 (0.37–1.84)	30/64 (46.9)	1.76 (0.88–3.56)	1.96 (0.95–4.04)	6/64 (9.4)	0.38 (0.14–1.07)	0.25 (0.08–0.79)
Q2 (8.7 < GWG < 10.5)	2/68 (2.9)	0.39 (0.07–2.07)	0.40 (0.07–2.23)	12/68 (17.7)	0.53 (0.23–1.18)	0.54 (0.24–1.24)	20/68 (29.4)	0.83 (0.40–1.72)	0.85 (0.41–1.77)	7/67 (10.5)	0.43 (0.16–1.15)	0.37 (0.13–1.06)
Q3 (10.6 < GWG < 12.0)	5/69 (7.3)	Ref	Ref	20/69 (29.0)	Ref	Ref	23/69 (33.3)	Ref	Ref	14/66 (21.2)	Ref	Ref
Q4 (12.2 < GWG < 13.5)	6/55 (10.9)	1.57 (0.45–5.44)	1.90 (0.50–7.30)	15/55 (27.3)	0.92 (0.42–2.02)	0.98 (0.43–2.20)	22/55 (40.0)	1.33 (0.64–2.78)	1.35 (0.64–2.88)	6/54 (11.1)	0.46 (0.17–1.31)	0.62 (0.21–1.88)
Q5 (13.8 < GWG)	5/62 (8.1)	1.12 (0.31–4.08)	1.08 (0.28–4.12)	23/62 (37.1)	1.44 (0.69–3.00)	1.50 (0.71–3.18)	20/62 (32.3)	0.95 (0.46–1.98)	0.91 (0.43–1.93)	10/57 (17.5)	0.79 (0.32–1.95)	0.90 (0.34–2.41)
		p for trend: 0.033	p for trend: 0.43		p for trend: 0.53	p for trend: 0.55		p for trend: 0.325	p for trend: 0.213		p for trend: 0.207	p for trend: 0.029

a: Association between GWG and overweight was assessed based on 308 women due to ten missing data on mother's current BMI.

b: Adjusted by height, pre-pregnancy body mass index, parity at pregnancy, age at delivery, and period from delivery to current status.

GWG: Gestational weight gain during pregnancy, OR: Odds ratio, PR: Prevalence, Ref.: Reference.

## Discussion

Our historical cohort study using MCHH data of Japanese women demonstrated a significant linear association between women’s GWG during pregnancy and risks of chronic diseases, such as DM, hypertension, and being overweight, in later life. Furthermore, although a significant association between GWG and DM remained after adjustment for current BMI, the association between GWG and HTN was attenuated. To our best knowledge, this is the first study on the association of GWG with the risk of maternal chronic disease later in life.

This study found significant positive linear associations between increased GWG during pregnancy and the risks of hypertension and overweight in the women’s later lives, similar to previous studies^7–9^. A possible explanation for this association might be that women with excessive GWG due to systemic edema tend to be accompanied by preeclampsia, which is associated with higher risk for hypertension in later life^2^. However, our study also showed that this significant linear association was attenuated after adjusting for the current BMI, suggesting that obesity has an important mediating role on the significant association between GWG and hypertension in later life. That is, the women who had excessive GWG tended to have weight retention after delivery, leading to obesity in later life resulting in a higher risk for hypertension. This suggestion may be plausible as obesity is one of the major risk factors of chronic hypertension due to several etiologies, such as activation of the renin–angiotensin–aldosterone system, insulin resistance, inflammation, and dyslipidemia^11^. Although further studies may be needed to confirm our findings, this study suggests women with higher GWG should take care of obesity and hypertension in later life.

This study also showed that women with higher GWG had a significantly increased risk for DM in later life, which remained after adjustment for current BMI, indicating that the association was at least partially independent from BMI in later life, although DM was significantly associated with current obesity status^12^. Weight gain during pregnancy can be directly attributed to increases in the feto–placental unit (fetus, placenta, amniotic fluid, and gestational uterus), blood volume, and breast tissue as well as to metabolic changes that occur to increase the accumulation of water, fat, and protein in the cells of the mother. Most additional weight gain beyond these factors is attributed to the accumulation of maternal fat^13^. Diet and physical activity affect weight gain during pregnancy^14^, suggesting that higher GWG may be a proxy for habitual overeating and/or a lack of exercise, which are risk factors for diabetes^15^. Another possible hypothesis is that pre-existing higher insulin resistance not only exacerbates GWG but also increases future risk of diabetes. We believe that this finding of an independent association between higher GWG and diabetes in later life is reliable.

Our study did not find a significant association between GWG and future hyperlipidemia. Hyperlipidemia is caused not only by hereditary factors but also by many various habitual factors such as obesity, uncontrolled diabetes, smoking, and excessive alcohol consumption^16^. Our result suggested hyperlipidemia may strongly associate with such habitual factors than GWG.

Our study demonstrated that women with higher GWG during pregnancy had higher risks for diabetes, hypertension, and being overweight in later life. Although those diseases are important risk factors for further severe complications, such as heart failure, ischemic stroke, intracerebral hemorrhage, ischemic heart disease, and chronic kidney disease^17–21^, regular health checkups, and lifestyle interventions after the postpartum period may improve women’s long-term health. Further studies on intervention for higher GWG women are warranted.

Our study had several strengths. First, the results suggested that weight measurements, as documented by standard and inexpensive examinations at regular maternal checkups, are useful for predicting the future risk for chronic disease, and potentially could lead to better maternal health worldwide, even in countries with few medical resources. Second, because the duration from delivery to questionnaire completion was 35.8 years, this study assessed data over a very long period, which might have minimized bias and underestimation of the cumulative incidence of later disease. Third, because we could gather precise maternal data, such as body weight gain and prepregnancy BMI, recorded in the MCHH for each woman, recall bias was probably negligible.

On the other hand, there were several study limitations that should be considered. First, we were not able to assess the history of GDM as a confounding factor due to a lack of data in the MCHH, although a history of GDM was associated with DM as an outcome^22^. Second, although this study demonstrated that GWG during pregnancy had significant linear associations with increased risks of hypertension and DM in later life, a significant association was not detected in each category. This may have been because of the smaller sample size. As we were not able to calculate the minimum sample size for this study due to a lack of previous studies, further studies involving larger sample sizes are warranted. Third, the demographic and chronic disease data were obtained from the self-reported questionnaires, which were not validated. Thus, the diagnostic criteria, and treatment might have varied, so careful interpretation of our findings is needed. Finally, the age of onset of the outcomes (DM, hypertension, and hyperlipidemia) was not considered, although this may be an important bias. To assess this association more accurately, further studies using datasets that include onset age are needed.

In conclusion, higher GWG during pregnancy in non-overweight women was found to be significantly associated with increased risks of future maternal diabetes and hypertension.

## Materials and methods

This historical cohort study was conducted at the National Center for Child Health and Development (NCCHD) in Tokyo between April 2017 and November 2020^10^. We recruited participants through their daughters who were visiting the NCCHD for pregnancy checkups. The participants were asked to provide their MCHH in which their pregnancy course when carrying their daughters (when they were fetuses) had been recorded, and the researchers extracted the data. The participants also were asked to provide a self-reported questionnaire on their current health conditions, including DM, hypertension, and hyperlipidemia, as well as their current height and weight. MCHH and the questionnaire were provided through their daughters or by mail. In Japan, the MCHH, the data of which is usually recorded by obstetricians and midwives at each checkup, is the official tool for recording maternal demographic data, including prepregnancy body mass index (BMI), and GWG at each prenatal visit.

Women who agreed to participate and provide both their MCHH and questionnaire about their medical conditions were eligible in this study. Women with missing MCHH maternal demographics data, including age at delivery, and parity or GWG during pregnancy were excluded. Women with prepregnant BMI > 25 kg/m^2^ were also excluded as they have an obviously higher risk for DM, hypertension, and hyperlipidemia^23,24^. The study protocol was approved by the Institutional Review Board of the National Center for Child Health and Development in May 2019 (No. 197) and performed in accordance with the guidelines of the Declaration of Helsinki and other nationally valid regulations. Written informed consent was obtained from all participants.

The exposure of interest was maternal GWG during pregnancy, as documented in the MCHH. The participants were categorized according to their GWG into three groups as insufficient GWG, adequate GWG, and excessive GWG. Here, adequate GWG was defined according to the current Japanese national guideline^25^, which is stratified by prepregnant BMI status as follows: 12 kg ≤ GWG < 15 kg is adequate among women with prepregnant BMI < 18.5 kg/m^2^, and 10 kg ≤ GWG < 13 kg is adequate among women with prepregnant 18.5 kg/m^2^ ≤ BMI < 25 kg/m^2^. GWG less than and greater than adequate GWG were defined as insufficient GWG and excessive GWG, respectively. The outcomes of interest were current maternal health conditions, including DM, hypertension, hyperlipidemia, and being overweight, which was defined as 25 kg/m^2^ ≤ BMI according to a self-report questionnaire^26^. Possible responses to the questions on the current status of DM, hypertension, and hyperlipidemia of women were the following: (1) never diagnosed; (2) ever diagnosed, without/never routine checkup; (3) ever diagnosed, with routine checkup and without any intervention; and (4) ever diagnosed, with routine checkup, and some intervention. We considered women with any of the last three answers to have DM, hypertension, and hyperlipidemia.

All statistical analyses were performed with the statistical software package Stata SE 16 (STATA Corp., College Station, TX, USA). Each result was presented as an odds ratio (OR) and a 95% confidence interval. Values of *P* < 0.05 were defined as indicating statistical significance, and all statistical tests were two-tailed.

## Data Availability

Restrictions of the ethics committee at the National Center for Child Health and Development prohibit the authors from making the minimal data set publicly available. Data are available from the National Center for Child Health and Development for researchers who meet the criteria for access to confidential data. Requests for the data can be sent to ogawa-k@ncchd.go.jp.

## References

[1] Pace R, Brazeau AS, Meltzer S, Rahme E, Dasgupta K (2017). Conjoint associations of gestational diabetes and hypertension with diabetes, hypertension, and cardiovascular disease in parents: A retrospective cohort study. Am. J. Epidemiol..

[2] Dall'Asta A (2021). Cardiovascular events following pregnancy complicated by pre-eclampsia with emphasis on comparison between early- and late-onset forms: Systematic review and meta-analysis. Ultrasound Obstet. Gynecol..

[3] Hedderson MM, Gunderson EP, Ferrara A (2010). Gestational weight gain and risk of gestational diabetes mellitus. Obstet. Gynecol..

[4] Gibson KS, Waters TP, Catalano PM (2012). Maternal weight gain in women who develop gestational diabetes mellitus. Obstet. Gynecol..

[5] Truong YN, Yee LM, Caughey AB, Cheng YW (2015). Weight gain in pregnancy: Does the Institute of Medicine have it right?. Am. J. Obstet. Gynecol..

[6] Kominiarek MA (2018). Association between gestational weight gain and perinatal outcomes. Obstet. Gynecol..

[7] Smith DE, Lewis CE, Caveny JL, Perkins LL, Burke GL, Bild DE (1994). Longitudinal changes in adiposity associated with pregnancy. Coronary artery risk development in young adults study. JAMA.

[8] Hutchins F (2021). Gestational weight gain and long-term maternal obesity risk: A multiple-bias analysis. Epidemiology.

[9] Nehring I, Schmoll S, Beyerlein A, Hauner H, von Kries R (2011). Gestational weight gain and long-term postpartum weight retention: A meta-analysis. Am. J. Clin. Nutr..

[10] Hosoya S, Ogawa K, Morisaki N, Okamoto A, Arata N, Sago H (2023). Gestational glycosuria, proteinuria, and borderline hypertension in pregnancy are predictors for the later onset of maternal chronic disease. J. Obstet. Gynaecol. Res..

[11] Hall JE, de Carmo JM, de Silva AA, Wang Z, Hall ME (2015). Obesity-induced hypertension: interaction of neurohumoral and renal mechanisms. Circ. Res..

[12] Sullivan PW, Morrato EH, Ghushchyan V, Wyatt HR, Hill JO (2005). Obesity, inactivity, and the prevalence of diabetes and diabetes-related cardiovascular comorbidities in the U.S. 2000–2002. Diabetes Care.

[13] Champion ML, Harper LM (2020). Gestational weight gain: Update on outcomes and interventions. Curr. Diab. Rep..

[14] Teede HJ (2022). Association of antenatal diet and physical activity-based interventions with gestational weight gain and pregnancy outcomes: A systematic review and meta-analysis. JAMA Intern. Med..

[15] Stumvoll M, Goldstein BJ, van Haeften TW (2005). Type 2 diabetes: principles of pathogenesis and therapy. Lancet..

[16] Vodnala D, Rubenfire M, Brook RD (2012). Secondary causes of dyslipidemia. Am. J. Cardiol..

[17] Yamazaki D, Hitomi H, Nishiyama A (2018). Hypertension with diabetes mellitus complications. Hypertens. Res..

[18] Hamrahian SM, Falkner B (2017). Hypertension in chronic kidney disease. Adv. Exp. Med. Biol..

[19] Lakkis JI, Weir MR (2018). Obesity and kidney disease. Prog. Cardiovasc. Dis..

[20] Haslam DW, James WP (2005). Obesity. Lancet..

[21] Galicia-Garcia U (2020). Pathophysiology of type 2 diabetes mellitus. Int. J. Mol. Sci..

[22] Vounzoulaki E, Khunti K, Abner SC, Tan BK, Davies MJ, Gillies CL (2020). Progression to type 2 diabetes in women with a known history of gestational diabetes: Systematic review and meta-analysis. BMJ..

[23] Luo J, Hodge A, Hendryx M, Byles JE (2020). Age of obesity onset, cumulative obesity exposure over early adulthood and risk of type 2 diabetes. Diabetologia.

[24] Buscot MJ (2018). Distinct child-to-adult body mass index trajectories are associated with different levels of adult cardiometabolic risk. Eur. Heart J..

[25] Morisaki N (2021). Gestational weight gain growth charts adapted to Japanese pregnancies using a Bayesian approach in a longitudinal study: The Japan Environment and Children's Study. J. Epidemiol..

[26] Yazawa A (2020). Accuracy of self-reported weight, height and body mass index among older people in Japan. Geriatr. Gerontol. Int..

